# Technostress and academic motivation: direct and indirect effects on university students' psychological health

**DOI:** 10.3389/fpsyg.2023.1211134

**Published:** 2023-06-30

**Authors:** Federica Vallone, John Galvin, Maria Francesca Cattaneo Della Volta, Athfah Akhtar, Stephanie Chua, Emilie Ghio, Theodoros Giovazolias, Zoe Kazakou, Marina Kritikou, Katerina Koutra, Sanja Kovacevic, Geraldine Lee-Treweek, Ivana Mašková, Eirini Mavritsaki, Jelena Nastic, Michala Plassova, Iva Stuchlíková, Maria Clelia Zurlo

**Affiliations:** ^1^Dynamic Psychology Laboratory, Department of Political Sciences, University of Naples Federico II, Naples, Italy; ^2^Department of Humanities, University of Naples Federico II, Naples, Italy; ^3^Department of Psychology, University of Warwick, Coventry, United Kingdom; ^4^Birmingham City University, Birmingham, United Kingdom; ^5^Department of Psychology, School of Social Sciences, University of Crete, Crete, Greece; ^6^Western Balkans Institute, Belgrade, Serbia; ^7^Department of Psychology, Faculty of Education, University of South Bohemia, Ceské Budějovice, Czechia

**Keywords:** academic motivation, information and communication technologies, mediating effects, protective factors, psychological health, risk factors, technostress, university students

## Abstract

**Introduction:**

Research has well demonstrated that the pandemic entailed several implications among university students worldwide in terms of increased use of Information and Communication Technologies (ICTs), technostress, disruptions in academic goals and motivation processes, and growing psychological suffering. Responding to the new research need to go in-depth into the processes linking technostress and motivation dimensions to inform current research/interventions, the present study aimed to explore the direct effects of perceived Technostress dimensions (Techno-Overload, Work-Home Conflict, Pace of Change, Techno-Ease, Techno-Reliability, and Techno-Sociality) and Academic Motivation dimensions (Amotivation, Intrinsic, and Extrinsic Motivation dimensions) on students' perceived levels of Anxiety/Depression and test the potential indirect effect (mediating role) of Academic Motivation dimensions in the associations between Technostress and psychological health conditions.

**Methods:**

Overall, 1,541 students from five European countries (Czech Republic, Greece, Italy, Serbia, United Kingdom) completed a survey comprising a Background Information Form, the Technostress Scale, the Academic Motivation Scale-College, and the Hospital Anxiety and Depression Scale. Hayes' PROCESS tool was used to test direct and indirect (mediating) effects.

**Results:**

Data revealed that Techno-Overload, Work-Home Conflict, Amotivation, and Extrinsic Motivation-Introjected had a direct negative effect, whereas Techno-Ease, Techno-Reliability, Techno-Sociality, all Intrinsic Motivation dimensions, and Extrinsic Motivation-Identified had a direct protective role for students' psychological health. The significant indirect role of motivation dimensions in the associations between Technostress dimensions and Anxiety/Depression was fully supported.

**Discussion:**

Findings allow gaining further insight into the pathways of relationships between technostress, motivation, and psychological health, to be used in the current phase, featured by the complete restoration of face-to-face contacts, to inform the development of tailored research and interventions, which address lights and shadows of the technology use, and which take into account the necessity to enhance its potentials yet without impairing students' motivation and psychological health.

## 1. Introduction

University students are recognized globally as a population vulnerable to poor wellbeing (Zivin et al., 2009; Auerbach et al., 2018). Indeed, research conducted worldwide has highlighted remarkable rates of severe psychological disease, in particular anxiety and depression, which were substantially higher than those reported among the general population (Eisenberg et al., 2007; Ibrahim et al., 2013; Quek et al., 2019; Mavrandrea and Giovazolias, 2022).

The school-to-college transition typifies one pivotal shift, in terms of increased personal duties and responsibilities as well as new financial, social, and relational needs and demands (Galvin et al., 2020; Parker et al., 2021). Moreover, whether several lifetime mental disorders have first onset around emerging adulthood—that is the more common age of beginning college/university (Kessler et al., 2005; Giovazolias et al., 2010)—the psychological suffering and the severity of symptoms may be even exacerbated due to the concerns and perceived pressures about academic life, performance/success, and future plans (Beiter et al., 2015).

Noteworthy, the number of university students with a serious mental illness has risen globally during the COVID-19 pandemic (Browning et al., 2021; Charles et al., 2021; Gritsenko et al., 2021; Xu et al., 2021), which has imposed key changes and further challenges in their daily life (Aristovnik et al., 2020; Zurlo et al., 2020), resulting in declining levels of motivation and difficulties in self-regulation (Means et al., 2020; Gonzalez-Ramirez et al., 2021; Hicks et al., 2021; Tasso et al., 2021; Usher et al., 2021; Corpus et al., 2022), growing rates of stress and difficulties in concentrating (Son et al., 2020; Baltà-Salvador et al., 2021; Somma et al., 2021; Zurlo et al., 2022a,b), and increased anxiety and depression (Cao et al., 2020; Husky et al., 2020; Rusch et al., 2021).

Recent research has warned that several students are still experiencing difficulties in re-adjusting to the new circumstances, reporting increases in perceived stress linked to technology use, and weakening of in-person relational and social abilities, apathy, disengagement, as well as decreased focus, motivation, and psychological health (Parker et al., 2021; Caron et al., 2022; Corpus et al., 2022; Curelaru et al., 2022; Singh et al., 2022; Stoian et al., 2022).

Accordingly, there is an urgent need to provide updated research accounting for the impact this prolonged condition may have left. The present study therefore will target university students and seek to provide evidence that could foster interventions promoting their psychological health in the post-emergency time, featured by the complete restoration of in-presence courses and face-to-face contacts. This is by investigating on direct and indirect effects of two key variables, namely technostress dimensions and academic motivation dimensions, on students' anxious and depressive symptomatology.

### 1.1. Technostress and psychological health among university students

Technostress is a term defined by Brod (1984) to describe the human cost of the technological revolution, namely the effects—in terms of psychophysical health outcomes—of the perceived difficulties in dealing with, and adjusting to, the ICTs use.

Based on a multidimensional and transactional approach to stress (Lazarus and Folkman, 1984), several studies have identified and categorized different Technostress dimensions, namely Techno-Overload, Work-Home Conflict, Pace of Change, Techno-Ease, Techno-Reliability, and Techno-Sociality (Moore and Benbasat, 1991; Moore, 2000; DeLone and McLean, 2003; Ayyagari et al., 2011; Tarafdar et al., 2011; Kemp et al., 2019).

Specifically, *Techno-Overload* (i.e., the perception of being under pressure, forced to work faster, and longer due to the use of ICTs), *Work-Home Conflict* (i.e., the perception of lack of boundaries between work/study and private life due to the use of ICTs), and *Pace of Change* (i.e., the perception of frequent ICT-related changes and updates) have been considered as significant risk factors able to substantially exacerbate psychological suffering. Conversely, *Techno-Ease* (i.e., the perception of easiness in the use of ICTs to reach the desired outcomes), *Techno-Reliability* (i.e., the perception of trustworthiness of ICTs to carry out the desired activities), and *Techno-Sociality* (i.e., the perception of the use of ICT as a social communication tool, so that individuals can reach or be reached by other people from a distance and at any time) have been considered protective factors that foster adjustment and wellbeing (Ayyagari et al., 2011; Tarafdar et al., 2011; Galvin et al., 2022).

The effects of technology use in terms of individual, relational, and social wellbeing have been highly debated within international research in terms of both lights and shadows (Berg-Beckhoff et al., 2017; Vilhelmson et al., 2017; Charalampous et al., 2019; Baumeister et al., 2021). Indeed, ICTs use may simultaneously entail not only risks (e.g., techno-overload, misuse/abuse of technology, invasion of privacy, difficulties in planning time for academic activities, excessively relying on technology for social life rather than for face-to-face interactions, difficulties in “disconnecting” from the virtual world) but also resources (e.g., socialization, collaboration, exchanging of information/advice/support; connections to others beyond time/space boundaries, flexibility, time-saving) (Wellman et al., 2001; Haythornthwaite, 2005; Gemmill and Peterson, 2006; Suhail and Bargees, 2006; Ragu-Nathan et al., 2008; Chayko, 2014; Brivio et al., 2018; Kemp et al., 2019; Lattie et al., 2019; Dietz et al., 2020; Thomas et al., 2020; Borle et al., 2021; Kniffin et al., 2021).

Undoubtedly, the COVID-19 pandemic has added complexity to the international debate on risks and resources linked to ICTs, due to their prolonged, extensive, and almost exclusive use to maintain social/relational life (Aguilera-Hermida, 2020; Garfin, 2020; Papouli et al., 2020; Kniffin et al., 2021). This was particularly true in the educational and academic contexts (Panisoara et al., 2020), which was already featured by significant and growing changes and pressures in recent decades (Zurlo et al., 2016; European Commission, 2017, 2020) and—afterward—among the most deeply impacted sector by the pandemic (Plakhotnik et al., 2021).

In particular, within a period of creeping technological revolution, the onset of the pandemic resulted in academic activities being abruptly shifted to online platforms, and technology use increased quantitatively and changed qualitatively (Garfin, 2020; Papouli et al., 2020; Sundarasen et al., 2020; Browning et al., 2021; Kniffin et al., 2021). Indeed, students were required to spend a greater and prolonged amount of time per day online/using technological devices (i.e., blue light exposure) (Browning et al., 2021; Gruba et al., 2021; Hagedorn et al., 2021; Hosen et al., 2021; Mack et al., 2021; Reinhart et al., 2021; Yadav et al., 2021; Yu et al., 2021), which resulted in increased levels of perceived load, psychological suffering (Hussein et al., 2020; Al-Kumaim et al., 2021; Lemay et al., 2021; Malik and Javed, 2021; Morales-Rodriguez, 2021), and anxiety and depression (Sundarasen et al., 2020; Chinna et al., 2021; Denisov et al., 2021; Dirzyte et al., 2021; Gao et al., 2021; González-López et al., 2021; Xu and Wang, 2023). This was particularly harmful to those who were already considered problematic ICTs users, as they were forced to further increase their time “on screen” during the pandemic (Cai et al., 2021; Hosen et al., 2021; Islam et al., 2021; Xie et al., 2021).

Within this portrait, whether there is substantial evidence of the direct impact of Technostress dimensions on students' wellbeing (e.g., Nadeem et al., 2018; Abbas et al., 2020; Wang et al., 2020, 2021; Galvin et al., 2022), some recent studies have also underlined that the extensive technology use during the pandemic has also had a detrimental effect in terms of decrease in motivation as well as increase in apathy and disengagement (Parker et al., 2021; Corpus et al., 2022; Curelaru et al., 2022; Stoian et al., 2022), suggesting the need to explore the unique link between ICTs use and self-regulation/motivational processes in the current time.

### 1.2. Academic motivation and university students' psychological health

Motivation and self-regulation processes represent essential components for optimal human functioning and key aspects in students' life (Yoo and Marshall, 2022), determining academic success and wellbeing (Pisarik, 2009; Kotera et al., 2022; Mašková et al., 2022), in terms of performance (Ali, 2020; Tan, 2020) and psychological health (Ryan and Deci, 2000; Marler et al., 2021; Juntunen et al., 2022).

The self-determination theory (SDT; Deci and Ryan, 1985) represents one of the most recognized motivation theories globally and has been widely applied in research and interventions targeting the educational context (Deci et al., 1991; Müller and Louw, 2004; Ryan et al., 2006; Liu et al., 2016; Howard et al., 2021; Kritikou and Giovazolias, 2022). Within the self-determination theory, the experience of autonomy in motivation processes is defined as the extent to which people behave according to self-endorsed values. The regulation of behaviors can be situated along a continuum ranging from a complete lack of motivation and self-determination (i.e., amotivation) to high autonomy (i.e., internal regulation/intrinsic motivation), passing through high control (i.e., external regulation/extrinsic motivation) (Ryan and Deci, 2000). The more behaviors are regulated by autonomous motives, the more individuals will flourish and experience greater wellbeing. This hypothesis has been confirmed in several domains, including education (Ryan and Deci, 2000; Vansteenkiste et al., 2008).

Considering university students, based on the SDT, a specific measurement tool—namely the Academic Motivation Scale—College version (AMS-C; Vallerand et al., 1992)—has been developed and internationally adopted (e.g., Chong and Ahmed, 2012; Stover et al., 2012; Ardeńska et al., 2016; Zurlo et al., 2023). The AMS-C covers university students' Amotivation, three types of Intrinsic Motivation (i.e., Motivation To Know; Motivation Toward Accomplishment; Motivation To Experience Stimulation), and three types of Extrinsic Motivation (i.e., External Regulation; Introjected Motivation; Identified Motivation), allowing to address the multidimensionality of the theoretical framework.

In detail, Amotivation refers to a condition by which neither intrinsic nor extrinsic factors boost students' actions. Either they do not act or they act passively, as they feel incapable, powerless, and/or do not associate the link between their behavior and the expected outcomes. Students who are mainly amotivated are more likely to report poor academic outcomes, isolation/lowered sense of belonging to the university community, and reduced wellbeing (Vallerand et al., 1997; Baker, 2004; Ratelle et al., 2007; Marler et al., 2021).

On the opposite, at the highest level of autonomous functioning, intrinsic motivation describes students who perceive a sense of inherent enjoyment and pleasure from academic life (i.e., understanding new things; surpassing oneself; stimulating sensations). This results in feelings of freedom, satisfaction, and wellbeing (Ryan and Deci, 2000; Jie et al., 2022).

Finally, considering extrinsic motivation, some students may be mainly driven by external forces/pressures (typically from family and society) to enroll at university and to achieve academic success. These students may perform actions to receive rewards/prevent penalties in grades (i.e., external regulation) or to avoid feeling guilty or ashamed about being disloyal to, and/or incompliant with, others' expectations (i.e., introjected regulation). However, extrinsically motivated students may also perform actions that are accepted/recognized as personally valuable and meaningful (i.e., identified regulation), displaying a more autonomous regulation, better performance, and higher wellbeing (Liu et al., 2016).

Generally, there is clear evidence about the detrimental effect of amotivation, on the one hand, and positive effect of the more autonomous types of motivation, such as intrinsic motivation and identified regulation, on the other hand, on wellbeing and psychological health (Pisarik, 2009; Ryan and Deci, 2017; Kotera et al., 2022; Mašková et al., 2022). In contrast, evidence on the association between psychological outcomes and more controlled types of motivation, such as external and introjected regulation, is less straightforward. Whereas multiple studies have found a negative effect of controlled motivation on psychological outcomes (e.g., Pisarik, 2009; Ryan and Deci, 2017), there are also studies that show no such association (Kotera et al., 2022; Mašková et al., 2022).

Recent research has increasingly explored motivation from a multidimensional/transactional perspective, with particular reference to its mediating role within broader processes (Dana et al., 2021). In this direction, evidence suggests the mediating role of motivation in the relationship between academic self-efficacy and procrastination (Malkoç and Mutlu, 2018), parenting style and life satisfaction (Stavrulaki et al., 2021), personality types and social networking site addiction (Chen and Roberts, 2020), psychological needs and engagement/burnout (De Francisco et al., 2020), and situational job-related stressors and burnout (Rubino et al., 2009).

However, despite the abundance of research targeting students by focusing independently on technostress (Liu, 2010; Henderson et al., 2015; Lattie et al., 2019; Papouli et al., 2020) and academic motivation (Vallerand et al., 1992; Kritikou and Giovazolias, 2022; Kvintova et al., 2022; Mašková et al., 2022), to the best of our knowledge, research exploring the mediating role of academic motivation in the associations between technostress dimensions and psychological health is lacking. Yet, undoubtedly, the COVID-19 pandemic/containment measures and the current post-pandemic conditions have unveiled this fairly new research need.

### 1.3. The present study

Considering the literature and the research needs reported above, the present study aimed to test the direct effects of perceived Technostress dimensions (i.e., Techno-Overload, Work-Home Conflict, Pace of Change, Techno-Ease, Techno-Reliability, Techno-Sociality) and Academic Motivation dimensions (i.e., Amotivation; Intrinsic Motivation—To Know, Toward Accomplishment, Experience Stimulation; and Extrinsic Motivation—Identified, Introjected, External Regulation) on students' psychological health as measured by perceived levels of Anxiety and Depression, and the potential indirect effect (mediating role) of Academic Motivation dimensions in the associations between Technostress and psychological health conditions.

Specifically, taking into account the previously established effects of Technostress dimensions on students' wellbeing and, in particular, on the one hand, the negative impact of perceived stress linked to techno-overload, managing the pace of technological change, and weaker boundaries between work and home due to ICTs use, and, on the one other hand, the positive impact of perceived ICTs as easy, reliable, and helpful in being connected/communicate with others (e.g., Tarafdar et al., 2011; Abbas et al., 2020; Wang et al., 2021; Galvin et al., 2022), the following hypothesis has been tested:

*Hypothesis One* (H1): Technostress dimensions will be significantly related to university students' psychological health. Specifically, Techno-Overload, Work-Home Conflict, and Pace of Change will be significantly positively related to Anxiety and Depression (H1.a) while Techno-Ease, Techno-Reliability, and Techno-Sociality will be significantly negatively related to Anxiety and Depression (H1.b).

Moreover, considering recent studies suggests that the extensive technology use during the pandemic has had an influence on self-regulation processes, impairing motivation, increasing apathy and disengagement (Parker et al., 2021; Corpus et al., 2022; Curelaru et al., 2022; Stoian et al., 2022), the following hypothesis has been examined:

*Hypothesis Two* (H2): Technostress dimensions will be significantly related to university students' Academic Motivation. Specifically, Techno-Overload, Work-Home Conflict, and Pace of Change will be significantly positively related to Amotivation (H2.a), while Techno-Ease, Techno-Reliability, and Techno-Sociality will be significantly negatively related to Amotivation (H2.b).

Furthermore, considering the well-demonstrated positive impact of more autonomous types of motivation and the negative impact of amotivation on wellbeing, along with the mixed evidence on the association between more controlled types of motivation and psychological health (e.g., Pisarik, 2009; Ryan and Deci, 2017; Kotera et al., 2022; Mašková et al., 2022), the following hypothesis has been developed and was tested:

*Hypothesis Three* (H3): Academic Motivation dimensions will be significantly related to university students' psychological health. Specifically, Amotivation will be significantly positively related to Anxiety and Depression (H3.a), while Intrinsic Motivation dimensions will be significantly negatively related to Anxiety and Depression (H3.b).

Finally, in line with the growing number of studies supporting the potential mediating role of motivation (e.g., Chen and Roberts, 2020; Stavrulaki et al., 2021), and given the new strict bond between ICTs use and academic motivation as one of the marks deriving from the pandemic (e.g., Parker et al., 2021; Corpus et al., 2022; Curelaru et al., 2022; Stoian et al., 2022), it is sound to hypothesize that academic motivation may—at least partially—explain the relationship between perceived technostress dimensions and psychological health among university students. The following hypothesis was, therefore, explored:

*Hypothesis Four* (H4): Academic Motivation dimensions will play as significant mediators in the associations between Technostress dimensions and university students' psychological health.

## 2. Materials and methods

### 2.1. Participants and sampling

The present cross-sectional and multi-national study raised in the context of a broader European Project (Masked for Blind Review). National surveys were made available online using Qualtrics platform and were widely disseminated in five European countries (i.e., Czech Republic, Greece, Italy, Serbia, United Kingdom) as part of the project. Data were collected over the period from March 2022 to December 2022. Students were asked to participate in the online survey v*ia* both institutional channels (e.g., academic mailing lists) and informal channels (e.g., social media groups), and they were given all the relevant information about the research project. The research was performed in accordance with the 1964 Helsinki Declaration and its later amendments or comparable ethical standards, and students were provided with all the information about the privacy policy (e.g., the treatment and the confidentiality of their data). The project was approved by the Ethical Committee of each institution involved. Overall, 2,227 university students accessed the Qualtrics survey; of those, 1,901 provided informed consent. However, 1,541 students completed the survey in all its parts and were included in the final dataset.

### 2.2. Measures

The questionnaire included a section on background information, along with validated measures for Technostress dimensions, Academic Motivation, and Psychological Health Outcomes.

#### 2.2.1. Background information

The background information section included single-item questions on Sex, Age (in years), Ethnicity, Number of people living in the household, Course of Study, and Employment status. In addition, daily time (in hours) in using ICTs was also asked.

#### 2.2.2. Technostress dimensions

Technostress Dimensions were assessed using the Technostress Scale (Ayyagari et al., 2011), which consists of 17 items on a 7-point Likert scale ranging from 1 (strongly disagree) to 7 (strongly agree) and divided into six subscales, namely Techno-Overload (three items; e.g., “I feel pressured due to ICTs”); Work-Home Conflict (three items, e.g., “Using ICTs blurs boundaries between my university/work life and my home life”); Techno-Ease (three items, e.g., “It is easy to get results that I desire from ICTs”); Techno-Reliability (three items, e.g., “ICTs behave in a highly consistent way”); Techno-Sociality (two items, e.g., “The use of ICTs enables others to have access to me”); Pace of Change (three items, e.g., “I feel that the way ICTs work changes often”). The scale has been adopted globally and is recognized as a statistically valid tool for assessing Technostress dimensions (e.g., Christ-Brendemühl and Schaarschmidt, 2020; Camacho and Barrios, 2022; Galvin et al., 2022). In the present study, confirmatory factor analysis revealed satisfactory goodness-of-fit indices for the original six-factor model: that is, comparative fit index (CFI) = 0.957; Tucker–Lewis index (TLI) = 0.944; goodness-of-fit index (GFI) = 0.996; Bentler–Bonett non-normed fix index (NNFI) = 0.944; Bentler–Bonett normed fix index (NFI) = 0.949; root mean square error of approximation (RMSEA) = 0.058; and standardized root mean square residual (SRMR) = 0.046. Cronbach's α and McDonald's ω values were also satisfactory (Supplementary Table 1).

#### 2.2.3. Academic motivation

Academic Motivation dimensions were assessed using the Academic Motivation Scale—College version (AMS-C; Vallerand et al., 1992), which consists of 28 items on a 7-point Likert scale ranging from 1 (Does not correspond at all) to 7 (Corresponds a lot) and divided into seven subscales, namely Amotivation (four items, e.g., “I once had good reasons for going to college; however, now I wonder whether I should continue”); Extrinsic Motivation—External Regulation (four items, e.g., “In order to obtain a more prestigious job later on”); Extrinsic Motivation—Introjected (four items, e.g., “Because of the fact that when I succeed in college I feel important”); Extrinsic Motivation—Identified (four items, e.g., “Because I think that a college education will help me better prepare for the career I have chosen”); Intrinsic Motivation—To Know (four items, e.g., “For the pleasure I experience when I discover new things never seen before”); Intrinsic Motivation—To Experience Stimulation (four items, e.g., “For the pleasure that I experience when I read interesting authors”); Intrinsic Motivation—Toward Accomplishment (four items, e.g., “Because college allows me to experience a personal satisfaction in my quest for excellence in my studies”). The scale is one of the main tools adopted and tested internationally, and its psychometric proprieties are widely demonstrated (e.g., Stover et al., 2012; Wilkesmann et al., 2012; Slezackova and Bobková, 2014; Vasić, 2019; Zurlo et al., 2023). In the present study, confirmatory factor analysis revealed adequate goodness-of-fit indices for the original seven-factor model: that is, CFI = 0.919; TLI = 0.907; GFI = 0.971; NNFI = 0.907; NFI = 0.908; RMSEA = 0.066; SRMR = 0.054. Moreover, Cronbach's α and McDonald's ω values were satisfactory (Supplementary Table 1).

#### 2.2.4. Psychological health outcomes: anxiety and depression

Psychological Health Outcomes were assessed in terms of Anxiety and Depression using the Hospital Anxiety and Depression Scale (HADS; Zigmond and Snaith, 1983), which consists of 14 items on a 4-point Likert scale divided into two subscales: Anxiety (seven items, e.g., “Worrying thoughts go through my mind”) and Depression (seven items, e.g., “I have lost interest in my appearance”). Anxiety and Depression scores were also converted into percentages, and a score of 11 was considered the cutoff point in order to define the perceived clinically relevant levels of symptoms (Zigmond and Snaith, 1983). The scale has been extensively adopted internationally, and its statistical validity is well-demonstrated (Costantini et al., 1999; Michopoulos et al., 2008; Bužgová et al., 2015; Ilic et al., 2021). In the present study, confirmatory factor analysis revealed satisfactory goodness-of-fit indices for the original two-factor model: that is, CFI = 0.936; TLI = 0.924; GFI = 0.975; NNFI = 0.924; NFI = 0.925; RMSEA = 0.060; SRMR = 0.045. In addition, Cronbach's α and McDonald's ω values were also satisfactory (Supplementary Table 1).

### 2.3. Data analysis

First, preliminary analyses were conducted. Specifically, descriptive statistics were carried out for background information, Technostress dimensions, Academic Motivation dimensions, and Psychological Health outcomes. Clinical levels of Anxiety and Depression were also calculated (cutoff = 11; Zigmond and Snaith, 1983). Moreover, preliminarily to hypotheses testing, Pearson's correlations were carried out between study variables. Therefore, direct and indirect effects were tested using Hayes' PROCESS tool for SPSS (Model 4; Preacher and Hayes, 2008; Hayes, 2017), which is an advanced regression-based approach. Following the four recommended steps for conducting mediation analyses, the following statistics were evaluated: (1) the effects of Technostress dimensions on Anxiety/Depression (H1); (2) the effects of Technostress dimensions on Academic Motivation dimensions (H2); (3) the effects of Academic Motivation dimensions on Anxiety/Depression (H3); (4) the effects of Technostress dimensions on Anxiety/Depression through Academic Motivation dimensions (H4). To verify the significance of the indirect effects, the Z Sobel test (Sobel, 1982) and bias-corrected bootstrapped test with 5,000 replications to ensure the 95% confidence interval were used (Hayes and Scharkow, 2013). Partner Country was used as control variable. All the statistical analyses were carried out using the Statistical Package for the Social Sciences (SPSS; version 21) and JAva Structural Program (JASP; version 0.17.1).

## 3. Results

Characteristics of participants are reported in Table 1. Moreover, considering clinically relevant cases, data revealed that 36.6% of students (*n* = 555) displayed clinical levels of Anxiety whereas 11.3% (*n* = 171) reported clinical levels of Depression.

**Table 1:** Background characteristics (*N* = 1.541).

**Characteristic**	**Value**
**Sex** ***n*** **(%)**	
Women	1.082 (70.2)
Men	432 (28.0)
Other	9 (0.6)
Prefer not to say	18 (1.2)
**Age in years** ***M (SD)***	
Age	22.36 (6.07)
**Ethnicity** ***n*** **(%)**	
White/Caucasian	1.368 (88.8)
Asian	64 (4.2)
Chinese	5 (0.3)
Black	23 (1.5)
Hispanic/Latino	12 (0.8)
Middle/Near Eastern	8 (0.5)
Mixed ethnicity	38 (2.5)
Other	13 (0.8)
Missing	10 (0.6)
**Number of people living in household** ***M*** **(*****SD)***	
Number of people	3.23 (1.61)
**Course of study** ***n*****(%)**	
Bachelors	1.211 (78.6)
Masters	290 (18.8)
PhD or equivalent	31 (2.0)
Other	9 (0.6)
**Employment** ***n*****(%)**	
Full-time	177 (11.5)
Part-time	438 (28.4)
Not employed	836 (54.3)
Other	88 (5.7)
Missing	2 (0.1)
**Number of daily hours using ICTs** ***M (SD)***	
Number of hours	6.75 (3.11)
Czech Republic	6.99 (3.13)
Greece	5.98 (2.76)
Italy	6.79 (3.16)
Serbia	6.15 (3.19)
United Kingdom	7.44 (3.12)

Table 2 illustrates the means, standard deviations, and findings from preliminarily Pearson's correlations among study variables. Data revealed statistically significant associations among study variables, providing evidence endorsing the testing of direct and indirect hypotheses. However, given the non-significance of the associations of both *Pace of Change* and *Extrinsic Motivation-External Regulation* with neither anxiety nor depression, these two variables were not included in the final analyses (direct and indirect hypotheses testing). This was decided due to the necessity to keep parsimony in statistical models.

**Table 2:** Means (M), standard deviations (SD), and Pearson's correlations among the study variables (*N* = 1.541).

	**M (SD)**	**1**	**2**	**3**	**4**	**5**	**6**	**7**	**8**	**9**	**10**	**11**	**12**	**13**	**14**	**15**
**Technostress dimensions**																
1. Techno-overload	10.13 (4.25)	1														
2. Work-home conflict	10.47 (4.55)	0.52^**^	1													
3. Techno-ease	15.97 (3.64)	−0.27^**^	−0.09^**^	1												
4. Techno-reliability	14.28 (3.48)	−0.29^**^	−0.12^**^	0.56^**^	1											
5. Techno-sociality	11.60 (2.27)	−0.07^**^	0.01	0.37^**^	0.37^**^	1										
6. Pace of change	13.72 (4.10)	0.15^**^	0.12^**^	0.03	0.00	0.13^**^	1									
**Academic motivation**																
7. Intrinsic motivation—to know	21.56 (5.55)	0.00	0.01	0.15^**^	0.13^**^	0.18^**^	0.06^*^	1								
8. Intrinsic motivation—toward accomplishment	17.64 (6.49)	−0.02	0.03	0.08^**^	0.11^**^	0.13^**^	0.16^**^	0.65^**^	1							
9. Intrinsic motivation—experience stimulation	16.65 (6.59)	0.05^*^	0.03	0.06^*^	0.08^**^	0.08^**^	0.10^**^	0.72^**^	0.61^**^	1						
10. Extrinsic motivation—identified	21.64 (5.45)	−0.01	0.01	0.13^**^	0.18^**^	0.19^**^	0.11^**^	0.47^**^	0.46^**^	0.38^**^	1					
11 Extrinsic motivation—introjected	18.22 (6.47)	0.03	0.07^**^	0.04	0.08^**^	0.13^**^	0.13^**^	0.31^**^	0.63^**^	0.26^**^	0.36^**^	1				
12. Extrinsic motivation—external regulation	19.72 (6.04)	0.01	0.05^*^	0.11^**^	0.16^**^	0.14^**^	0.07^**^	0.06^*^	0.20^**^	0.01	0.55^**^	0.39^**^	1			
13. Amotivation	7.44 (5.02)	0.13^**^	0.15^**^	−0.08^**^	−0.08^**^	−0.13^**^	0.03	−0.49^**^	−0.34^**^	−0.29^**^	−0.36^**^	−0.09^**^	−0.02	1		
**Psychological health outcomes**																
14. Anxiety	9.12 (4.54)	0.28^**^	0.25^**^	−0.16^**^	−0.19^**^	−0.05^*^	0.03	−0.09^**^	−0.08^**^	−0.06^*^	−0.03	0.13^**^	0.02	0.20^**^	1	
15. Depression	5.88 (3.71)	0.23^**^	0.24^**^	−0.12^**^	−0.17^**^	−0.11^**^	0.03	−0.23^**^	−0.19^**^	−0.15^**^	−0.16^**^	−0.02	−0.01	0.33^**^	0.60^**^	1

* *p* < 0.05;

** *p* < 0.01.

With respect to *Hypothesis One* (H1), Techno-Overload, and Work-Home Conflict were significantly positively related to Anxiety and Depression (H1.a), while Techno-Ease, Techno-Reliability, and Techno-Sociality were significantly negatively related to Anxiety and Depression (H1.b).

With respect to *Hypothesis Two* (H2), Techno-Overload and Work-Home Conflict were significantly positively related to Amotivation (H2.a), and Work-Home Conflict was also significantly positively related to Extrinsic Motivation—Introjected.

Moreover, Techno-Ease, Techno-Reliability, and Techno-Sociality were significantly negatively related to Amotivation (H2.b), and they were also significantly positively related to all Intrinsic Motivation dimensions.

With respect to *Hypothesis Three* (H3), Amotivation was significantly positively related to Anxiety and Depression (H3.a), while all the Intrinsic Motivation dimensions were significantly negatively related to Anxiety and Depression (H3.b). Considering Extrinsic Motivation dimensions, Extrinsic Motivation—Identified was significantly negatively related to Depression while Extrinsic Motivation—Introjected was significantly positively related to Anxiety.

With respect to *Hypothesis Four* (H4), Academic Motivation dimensions acted as significant mediators in the associations between Technostress dimensions and university students' psychological health conditions. Table 3 shows path coefficients (direct and indirect effects) of Technostress dimensions and Academic Motivation on Anxiety/Depression.

**Table 3:** Path coefficients: direct and indirect effects of technostress dimensions and academic motivation on anxiety/depression.

**Independent variable**	**Mediator**	**Dependent variable**	**Path A^a^ (95% C.I.)**	**Path B^b^** **(95% C.I.)**	**Direct Effect**^c^ **(95% C.I.)**	**Indirect Effect^d^** **(95% C.I.)**	**Sobel's Z^e^**
Techno-overload	Amotivation	Anxiety^f^	0.17 (0.11, 0.22)^***^	14 (0.10, 0.19)^***^	28 (0.23, 0.33)^***^	02 (0.01, 0.04)^***^	4.23^***^
		Depression^f^	0.17 (0.11, 0.22)^***^	23 (0.20, 0.27)^***^	17 (0.13, 0.21)^***^	04 (0.02, 0.06)^***^	5.15^***^
Work-home conflict	Extrinsic motivation—introjected	Anxiety^f^	0.10 (0.03, 0.17)^***^	07 (0.04, 0.11)^***^	24 (0.19, 0.29)^***^	01 (0.00, 0.02)^***^	2.24^*^
	Amotivation	Anxiety^f^	0.15 (0.10, 0.21)^***^	15 (0.10, 0.19)^***^	23 (0.18, 0.27)^***^	02 (0.01, 0.04)^***^	4.25^***^
		Depression^f^	0.15 (0.10, 0.21)^***^	23 (0.20, 0.27)^***^	16 (0.12, 0.20)^***^	04 (0.02, 0.05)^***^	5.12^***^
Techno-ease	Intrinsic motivation—to know	Anxiety^f^	0.24 (0.17, 0.32)^***^	−0.05 (−0.09, −0.01)^*^	−0.20 (−0.26, −0.13)^***^	−0.01 (−0.02, −0.00)^*^	−2.14^*^
		Depression^f^	0.24 (0.17, 0.32)^***^	−0.14 (−0.18, −0.11)^***^	−0.09 (−0.14, −0.04)^***^	−0.04 (−0.05, −0.02)^***^	−5.12^***^
	Intrinsic motivation—toward accomplishment	Depression^f^	0.16 (0.07, 0.25)^***^	−0.10 (−0.13, −0.07)^***^	−0.11 (−0.16, −0.06)^***^	−0.02 (−0.03, −0.01)^**^	−3.15^**^
	Intrinsic motivation—experience stimulation	Depression^f^	0.15 (0.06, 0.23)^***^	−0.09 (−0.11, −0.06)^***^	−0.12 (−0.17, −0.07)^***^	−0.01 (−0.02, −0.00) ^**^	−2.88^**^
	Extrinsic motivation—identified	Depression^f^	0.21 (0.14, 0.28)^***^	−0.10 (−0.14, −0.07)^***^	−0.11 (−0.16, −0.06)^***^	−0.02 (−0.04, −0.01)^***^	−4.03^***^
	Amotivation	Anxiety^f^	−0.13 (−0.20, −0.06)^***^	0.16 (0.12, 0.21)^***^	−0.19 (−0.25, −0.13)^***^	−0.02 (−0.04, −0.01)^***^	−3.33^***^
		Depression^f^	−0.13 (−0.20, −0.06)^***^	0.24 (0.21, 0.28)^***^	−0.10 (−0.14, −0.05)^***^	−0.03 (−0.05, −0.02)^***^	−3.64^***^
Techno-reliability	Intrinsic motivation—to know	Anxiety^f^	0.24 (0.16, 0.32)^***^	−0.04 (−0.09, −0.00)^*^	−0.26 (−0.32, −0.19)^***^	−0.01 (−0.02, −0.00)^*^	−2.02^*^
		Depression^f^	0.24 (0.16, 0.32)^***^	−0.14 (−0.18, −0.11)^***^	−0.15 (−0.20, −0.09)^***^	−0.03 (−0.05, −0.02)^***^	−4.86^***^
	Intrinsic motivation—toward accomplishment	Depression^f^	0.24 (0.15, 0.34)^***^	−0.10 (−0.13, −0.07)^***^	−0.16 (−0.21, −0.11)^***^	−0.02 (−0.04, −0.01)^***^	−4.09^***^
	Intrinsic motivation—experience stimulation	Depression^f^	0.25 (0.16, 0.34)^***^	−0.08 (−0.11, −0.05)^***^	−0.16 (−0.22, −0.11)^***^	−0.02 (−0.03, −0.01)^***^	−3.77^***^
	Extrinsic motivation—introjected	Anxiety^f^	0.14 (0.15, 0.24)^***^	0.10 (0.06, 0.13)^***^	−0.28 (−0.35, −0.22)^***^	0.01 (0.00, 0.03)^**^	2.64^**^
	Extrinsic motivation—identified	Depression^f^	0.31 (0.24, 0.39)^***^	−0.09 (−0.13, −0.06)^***^	−0.15 (−0.22, −0.10)^***^	−0.03 (−0.04, −0.02)^***^	−4.43^***^
	Amotivation	Anxiety^f^	−0.15 (−0.22, −0.07)^***^	0.16 (0.12, 0.20)^***^	−0.25 (−0.31, −0.18)^***^	−0.02 (−0.04, −0.01)^***^	−3.46^***^
		Depression^f^	−0.15 (−0.22, −0.07)^***^	0.24 (0.21, 0.28)^***^	−0.15 (−0.20, −0.10)^***^	−0.03 (−0.05, −0.02)^***^	−3.83^***^
Techno-sociality	Intrinsic motivation—to know	Anxiety^g^	0.46 (0.34, 0.58)^***^	−0.06 (−0.10, −0.02)^**^	−0.09 (−0.19, 0.01)	−0.03 (−0.05, −0.01)^**^	−2.63^**^
		Depression^f^	0.46 (0.34, 0.58)^***^	−0.15 (−0.18, −0.11)^***^	−0.12 (−0.20, −0.04)^**^	−0.07 (−0.10, −0.04)^***^	−5.70^***^
	Intrinsic motivation—toward accomplishment	Anxiety^f^	0.38 (0.24, 0.53)^***^	−0.05 (−0.08, −0.01)^*^	−0.10 (−0.20, −0.00)^*^	−0.02 (−0.04, −0.00)^*^	−2.30^*^
	Techno-sociality	Depression^f^	0.39 (0.24, 0.53)^***^	−0.10 (−0.13, −0.07)^***^	−0.15 (−0.23, −0.07)^***^	−0.04 (−0.06, −0.02)^***^	−4.24^***^
	Intrinsic motivation—experience stimulation	Depression^f^	0.29 (0.16, 0.43)^***^	−0.08 (−0.11, −0.05)^***^	−0.16 (−0.24, −0.08)^***^	−0.02 (−0.04, −0.01)^***^	−3.35^***^
	Extrinsic motivation—introjected	Anxiety^f^	0.35 (0.21, 0.49)^***^	0.09 (0.06, 0.13)^***^	−0.15 (−0.25, −0.05)^**^	0.03 (0.02, 0.05)^***^	4.51^***^
	Extrinsic motivation—identified	Depression^f^	0.47 (0.35, 0.58)^***^	−0.10 (−0.14, −0.07^***^	−0.14 (−0.22, −0.06)^***^	−0.05(−0.07, −0.03)^***^	−4.62^***^
	Amotivation	Anxiety^g^	−0.31 (−0.42, −0.20)^***^	0.17 (0.13, 0.22)^***^	−0.06 (−0.16, 0.03)	−0.05 (−0.08, −0.03)^***^	−4.50^***^
		Depression^f^	−0.31 (−0.42, −0.20)^***^	0.24 (0.21, 0.28)^***^	−0.11 (−0.19, −0.03)^**^	−0.08 (−0.12, −0.04)^***^	−5.20^***^

Only significant mediation models were displayed. All the paths are controlled by partner country.

a Path A, effect of independent variable on mediator.

b Path B, effect of mediator on dependent variable.

c Direct effect, effect of independent variable on dependent variable controlling for the mediator.

d Indirect effect, effect of independent variable on dependent variable through the mediator.

e Sobel's *Z*, Sobel test results for indirect effect.

f Partial mediation.

g Full mediation.

* *p* < 0.05.

** *p* < 0.01.

*** *p* < 0.001.

Specifically, data highlighted the negative impact of Techno-Overload and Work-Home Conflict on Anxiety and Depression *via* Amotivation, as well as the negative impact of Work-Home Conflict on Anxiety *via* Extrinsic Motivation—Introjected. Differently, data enlightened the positive impact of Techno-Ease, Techno-Reliability, and Techno-Sociality on psychological health *via* Amotivation, *via* all Intrinsic Motivation dimensions as well as *via* Extrinsic Motivation—Identified.

**Figure 1:**
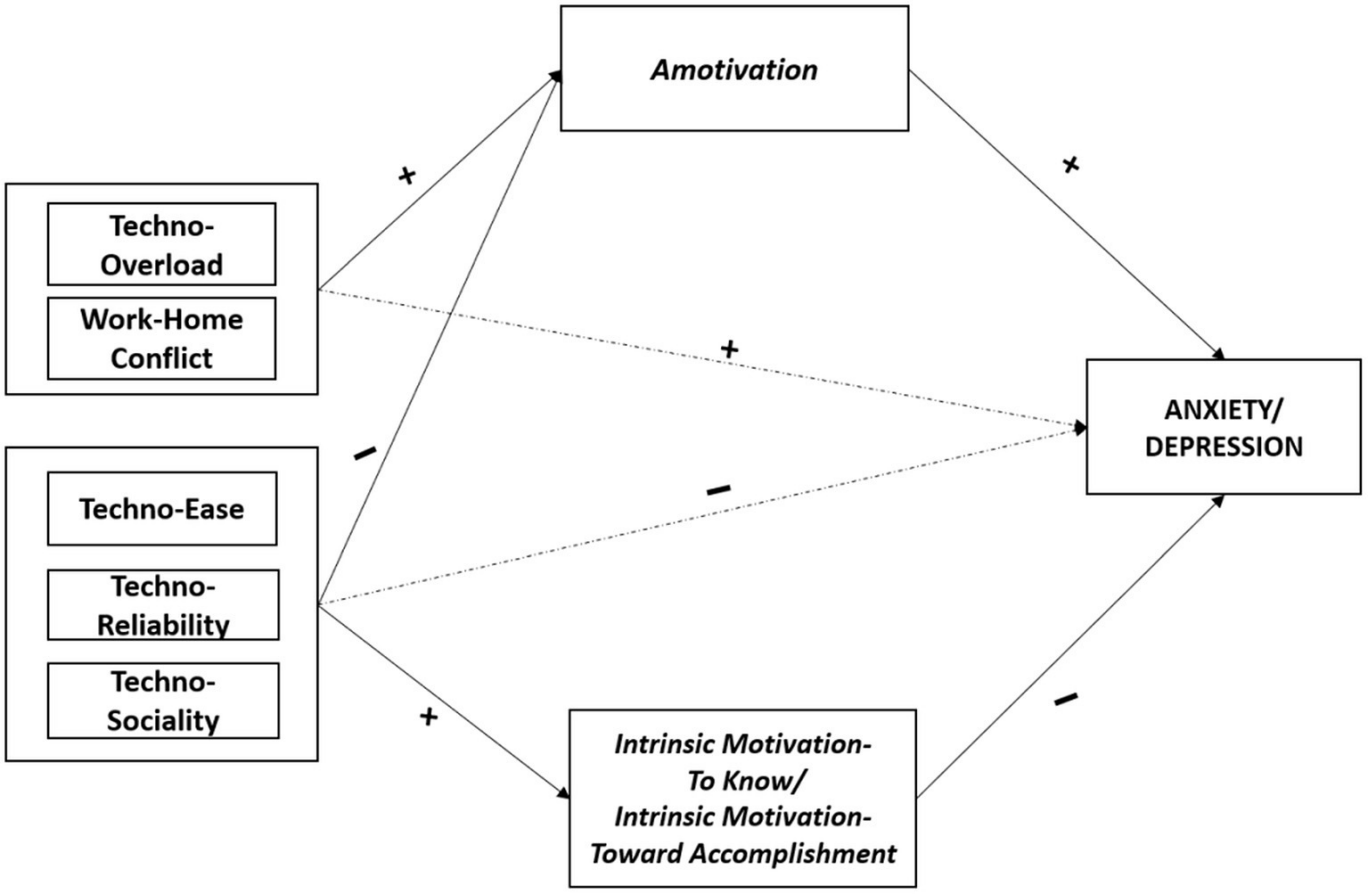
Summary—the mediating role of amotivation, intrinsic motivation—to know, and intrinsic motivation—toward accomplishment in the associations between technostress dimensions and anxiety/depression. Mediating variables are displayed in italics; psychological health outcomes are displayed in capital. Symbols (+, –) indicate the directions of the associations.

Figure 1 illustrates the indirect effect of Amotivation, Intrinsic Motivation—To Know, and Intrinsic Motivation—Toward Accomplishment in the associations between Technostress dimensions (Techno-Overload, Work-Home Conflict, Techno-Ease, Techno-Reliability, and Techno-Sociality) and Anxiety/Depression.

**Figure 2:**
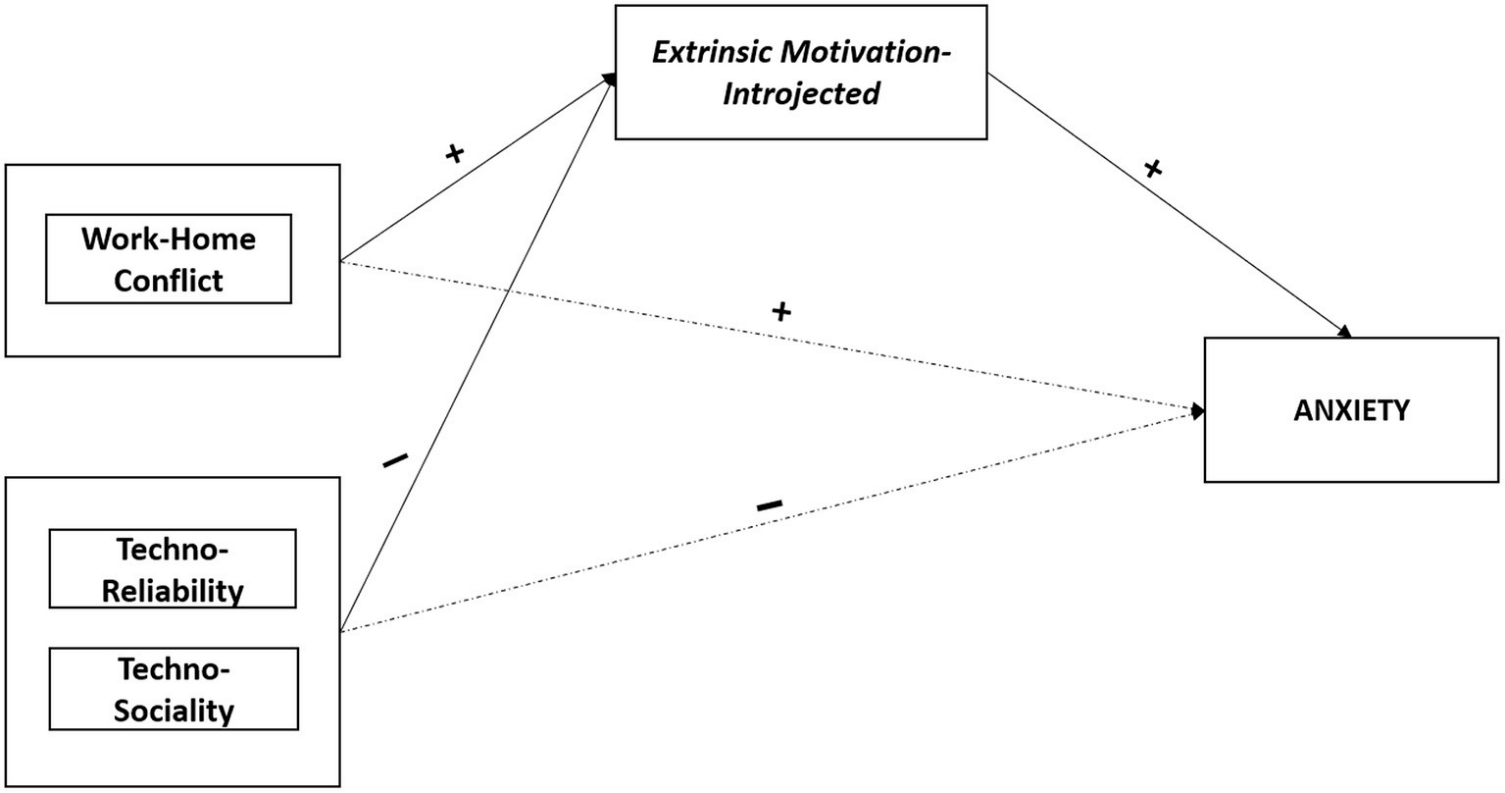
Summary—The mediating role of extrinsic motivation—introjected in the associations between technostress dimensions and anxiety. Mediating variables are displayed in italics; psychological health outcomes are displayed in capital. Symbols (+, –) indicate the directions of the associations.

Figure 2 shows the indirect effect of Extrinsic Motivation—Introjected in the associations between Technostress dimensions (Work-Home Conflict, Techno-Ease, Techno-Reliability, and Techno-Sociality) and Anxiety.

**Figure 3:**
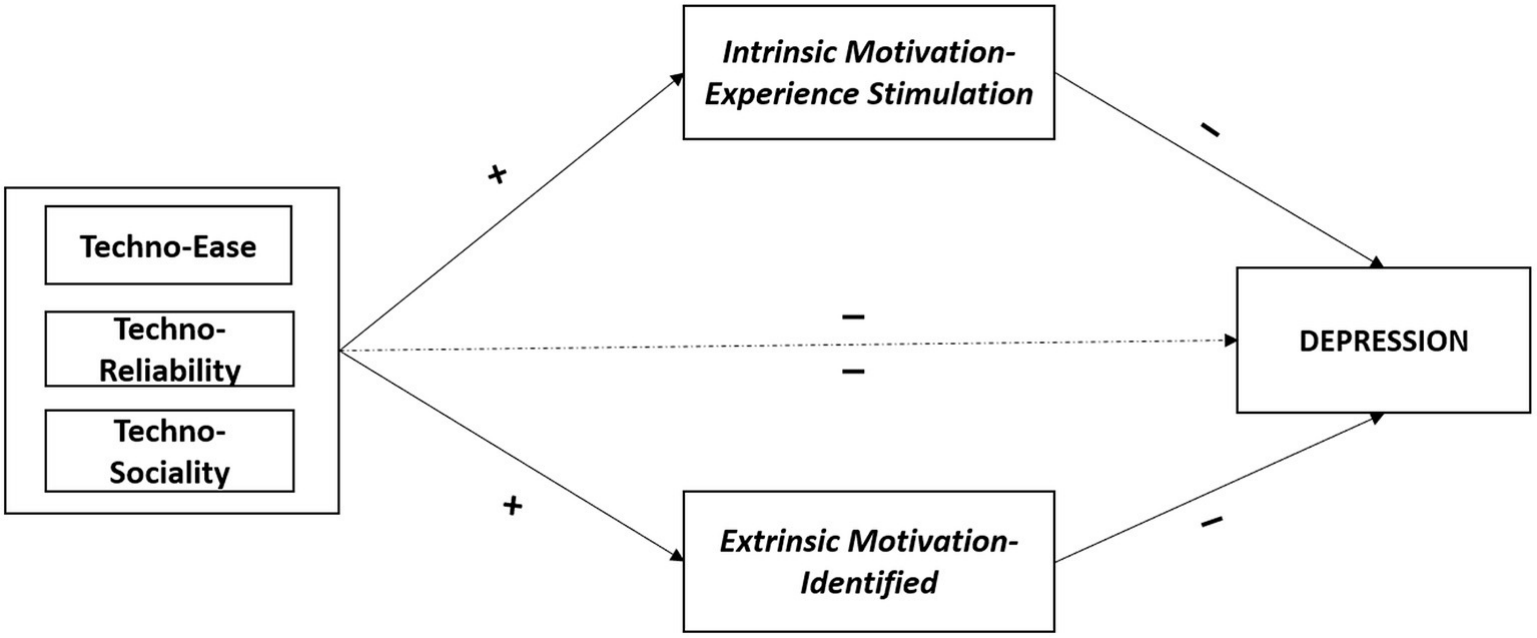
Summary—the mediating role of intrinsic motivation—experience stimulation and extrinsic motivation—identified in the associations between technostress dimensions and depression. Mediating variables are displayed in italics; psychological health outcomes are displayed in capital. Symbols (+, –) indicate the directions of the associations.

Figure 3 illustrates the indirect effect of Intrinsic Motivation—Experience Stimulation and Extrinsic Motivation—Identified in the associations between Technostress dimensions (Techno-Ease, Techno-Reliability, and Techno-Sociality) on Depression.

## 4. Discussion

The present multi-national study provides information on university students' experience at the current time, offering tailored indications on ICTs use and motivation processes, and fostering the understanding of the dimensions that may directly and/ or indirectly impact their psychological health conditions. This is also due to the need for timely identifying and supporting the great number of students who are still struggling in re-adjusting to the post-emergency condition and/or reported clinically relevant levels of Anxiety and Depression. In the present study, the remarkable number of students reporting clinically relevant levels of anxiety (about 40%) and depression levels (about 11%) regrettably sustains this need.

The current study provided updated evidence allowing us to go in-depth into the process linking technostress dimensions, academic motivation, and psychological health, so contributing to the international debate on the role of ICTs, in terms of risks but also of potential resources. As a result, the findings can help to inform evidence-based interventions effectively promoting students' wellbeing.

First, we found support for *Hypothesis One* (H1) and *Hypothesis Two* (H2), on the impact of Technostress dimensions (except for Pace of Change)—respectively—on students' psychological health (H1) and Amotivation (H2), in the expected directions. Moreover, considering H2, our findings highlighted further statistically significant associations, which—instead—were not hypothesized *a priori* due to the still lacking research in this field. Specifically, Work-Home Conflict was found significantly positively related to Extrinsic Motivation—Introjected, whereas Techno-Ease, Techno-Reliability, and Techno-Sociality were found to significantly positively relate to all Intrinsic Motivation dimensions. These data offered further evidence on the direct relationship between Technostress dimensions and Academic Motivation dimensions, fully endorsing the meaningfulness to test more complex pathways of associations among them. Moreover, these data corroborate with the international research evidence on the detrimental role of technology overuse/abuse/misuse (Thomée et al., 2007; Brooks, 2015; Brivio et al., 2018; Marler et al., 2021; Juntunen et al., 2022), as well as on the protective role of specific technology-related dimensions, in terms of Techno-Ease, Techno-Reliability, and Techno-Sociality (Saadé and Kira, 2009; Shah et al., 2012; Bower, 2019; Galvin et al., 2022).

Second, we found support for *Hypothesis three* (H3), again highlighting associations in the expected directions. These data were in line with evidence on the relationship between motivation/self-regulation processes and wellbeing (Ryan and Deci, 2000; Marler et al., 2021; Juntunen et al., 2022) and, in particular, the well-demonstrated negative role of Amotivation (Vallerand et al., 1997; Baker, 2004; Ratelle et al., 2007; Marler et al., 2021) and the role of Intrinsic Motivation dimensions as key resources (Ryan and Deci, 2000; Jie et al., 2022) for students' psychological health. However, when considering extrinsic motivation, we made no hypothesis on the direction of associations with anxiety/depression due to the mixed evidence reported in the literature. Our data revealed that higher levels of Extrinsic Motivation—Introjected were found to be associated with increased anxious symptoms, while higher levels of Extrinsic Motivation—Identified were associated with lower depressive symptoms.

These findings supported the need to promote, within the higher educational context, processes toward internalization, appropriation, and re-appropriation of the individual and autonomous motivation to enter and continue university. From this perspective, when extrinsically motivated, behaviors are controlled to obtain a reward/to avoid a constraint so that students perform actions mainly to fulfill social/familiar expectations. Accordingly, the experiences of external pressures to achieve academic success, together with the actual duties and challenges to be faced, may indubitably exacerbate students' concerns, worries, and anxiety. Differently, extrinsically motivated students, who display a more autonomous regulation, may have greater tools and resources to deal with academic demands, reporting lower psychopathological risk (Ryan and Deci, 2000; Liu et al., 2016).

Notwithstanding the interest in these results, the key finding from the present study concerns the evidence supporting *Hypothesis four* (H4), namely the mediating role (indirect effects) of Academic Motivation dimensions in the associations between Technostress dimensions and psychological health reported by university students. In line with research highlighting the mediating role of motivation within broader processes (Rubino et al., 2009; Malkoç and Mutlu, 2018; Chen and Roberts, 2020; De Francisco et al., 2020; Stavrulaki et al., 2021), this study provides original evidence on the underlying mechanisms linking ICTs use and Anxiety/Depression *via* Academic Motivation.

Considering the unique interplay between Technostress dimensions and Academic Motivation dimensions, our results underlined both vicious and virtuous circles that could be used for developing tailored support interventions addressing both lights and shadows of ICTs use. In particular, with respect to the process linking technology-related risk factors (i.e., Techno-Overload and Work-Home Conflict), Academic Motivation, and Psychological Health, data have highlighted the negative impact of Techno-Overload and Work-Home Conflict on Anxiety and Depression partially *via* Amotivation, as well as the negative impact of Work-Home Conflict on Anxiety partially *via* Extrinsic Motivation—Introjected. Therefore, high stress related to technological burden and conflict between academic/work and private life due to ICTs use may detriment students' psychological health also through the impairment of motivation and self-regulation process.

From this perspective, even after the end of the COVID-19 emergency, and as a key mark resulting from the prolonged containment measures, we believe that these data suggest that students who still rely excessively on technological devices in order to perform academic activities (e.g., use of online platforms to meet professors and social networks to stay in touch with colleagues) can also experience lowered motivation and high psychological suffering. This could be due to the increasing withdrawal from the university community by these students (Marler et al., 2021), often resulting in a perceived distance between their own experience and that of colleagues, and a lowered sense of autonomy over their own choices. Alongside, considering emerging adulthood (i.e., within an already complex transitional moment of growth and challenging path toward independence), the increasingly thin and blurred boundaries between academic/work and personal/family life due to the pandemic may have even hindered the possibility to accomplish internalizing processes of academic motivation. Accordingly, these students could be at higher risk of passively performing academic activities mainly to avoid feeling guilty or ashamed about being incompliant with family expectations (i.e., introjected motivation).

These underlined processes should be carefully considered when defining support interventions for students, due to the high risk of a vicious circle exacerbating anxiety (i.e., due to perceived growing social/family pressures to have success, fears to be left behind and performing worse than all the other students, concerns about the future), sense of helplessness, loneliness, and hopelessness (Beiter et al., 2015).

Considering protective factors linked to technology use (virtuous circles), the current study highlighted the positive impact of Techno-Ease, Techno-Reliability, and Techno-Sociality on psychological health *via* Amotivation, *via* all Intrinsic Motivation dimensions, and *via* the more autonomous extrinsic motivation factor, namely Extrinsic Motivation—Identified.

From this perspective, results confirmed previous research indicating that the perceived easiness of using ICTs and the perceived reliability of technological devices may represent important resources able not only to enhance performance (Bower, 2019) but also to promote individuals' wellbeing (Saadé and Kira, 2009; Shah et al., 2012). These findings provided evidence highlighting the need to furnish students with adequate information and tools to effectively use ICTs. Faculty members and university staff/authorities may, therefore, consider the meaningfulness to provide students with further resources and technical assistance to master challenges in technology use (Ragu-Nathan et al., 2008; Heckel and Ringeisen, 2019). Indeed, despite students being considered digital natives, they may still lack the theoretical knowledge required for particular skills, or have some limitations in their use of technology that could hinder their learning. This is particularly true considering that, following the emergency transition to distance learning, students were required to abruptly adapt to effectively use new platforms, and this may have increased their shame and sense of ineffectiveness when unable to use ICTs (Aguilera-Hermida, 2020; Sundarasen et al., 2020; Browning et al., 2021), potentially resulting in loss of motivation, withdrawals, and even leaving intentions. Conversely, when students perceive easiness in the use of ICTs as well as the trustworthiness of ICTs to reach the desired outcomes and to keep in touch with others this may result in a higher sense of autonomy, increased pleasure and enjoyment for academic paths and, therefore, result in higher psychological wellbeing.

Moreover, interestingly, whereas data suggested that academic motivation partially explains the relationship between the majority of Technostress dimensions with students' psychological health, findings on Techno-Sociality also revealed some full mediations. Specifically, the relationship between Techno-Sociality and Anxiety was fully mediated by both Intrinsic Motivation—To Know and Amotivation. These findings seem to suggest a more intimate link between the social and relational features of ICTs and motivational process and endorsed the idea that ICTs use should not be stigmatized in itself, as it can help students to stay active, connected, and engaged and, therefore, to report higher wellbeing. From this perspective, support interventions should carefully consider that ICTs can represent a key relational tool for students (Liu, 2010; Henderson et al., 2015; Lattie et al., 2019; Papouli et al., 2020; Thomas et al., 2020), but also a double-edged sword—without the required awareness (Wellman et al., 2001; Haythornthwaite, 2005; Gemmill and Peterson, 2006; Suhail and Bargees, 2006; Brivio et al., 2018; Kniffin et al., 2021).

Overall, these results recommend the importance of planning interventions accounting for the need to face students' difficulties in effectively using ICTs and in re-adjusting to in-person life, so disconnecting from the exclusive virtual world experienced for a prolonged time. In this direction, interventions should also take into account the need to support the building of a sense of academic community and social support networks (both face to face and by ICTs), in order to promote the development/restoration of students' active choice of their academic path, while reducing, at the same time, the risks—in terms of wellbeing—of the excessive use/misuse of technology.

Notwithstanding the potential strengths of the study, our findings should be interpreted also considering some methodological limitations. Firstly, the cross-sectional design of our study does not allow causal conclusions to be drawn. Moreover, the links between Technostress and Academic Motivation could also be bidirectional, may change over time, and may be not linearly related to the expression of psychopathology. Therefore, future research could be conducted with a longitudinal design to study the hetero-determination of the contextual relationship. Second, despite the sample comprising students from five European countries, the overall homogeneity of our sample, which predominately consisted of young and Caucasian students, limits the generalizability of our findings to the university students' population. In addition, despite the analyses being conducted controlling for partner country, so accounting for the potential impact of this factor, country specificities were not analyzed, requiring these findings to be interpreted and used with caution. Indeed, country differences were not the focus of the present study and further dimensions should be explored in future (e.g., socio-cultural factors, differences in the adoption of online learning pre-, during, and post-pandemic emergency, as well as differences in higher education systems). Nonetheless, despite the needed caution, these data could be used to develop research and support interventions within European countries.

In conclusion, despite these limitations, the study provides original evidence on the pathways of relationships between ICT use, motivation, and psychological health, to be used in the current phase, featured by the complete restoration of face-to-face contacts, to inform the development of tailored research and interventions fostering students' motivation and promoting their psychological health.

## Data availability statement

The raw data supporting the conclusions of this article will be made available by the authors, without undue reservation.

## Ethics statement

The studies involving human participants were reviewed and approved by the Ethical Committee of Psychological Research of the University of Naples Federico II (Protocol Code: 14/2022). The patients/participants provided their written informed consent to participate in this study.

## Author contributions

All authors contributed to the study design, revised the manuscript, and approved the final version.
