# Supplementary material for: Technostress and academic motivation: direct and indirect effects on university students' psychological health
Source: Front Psychol. 2023 Jun 30;14:1211134. doi: 10.3389/fpsyg.2023.1211134 (PMC10348917; doi:10.3389/fpsyg.2023.1211134)
Supplement: Supplementary file 1 [file Table_1.DOCX]

***Supplementary Table 1.*** *Cronbach’s α and* *McDonald’s ω values for the Technostress Scale, the Academic Motivation Scale-College and the Hospital Anxiety and Depression Scale.*

|  | **Total** | |  | **Czech Republic** | |  | **Greece** | |  | **Italy** | |  | **Serbia** | |  | **United**  **Kingdom** | |
| --- | --- | --- | --- | --- | --- | --- | --- | --- | --- | --- | --- | --- | --- | --- | --- | --- | --- |
|  | *α* | *ω* |  | *α* | *ω* |  | *α* | *ω* |  | *α* | *ω* |  | *α* | *ω* |  | *α* | *ω* |
| **Technostress Scale** |  |  |  |  |  |  |  |  |  |  |  |  |  |  |  |  |  |
| Techno-Overload | 0.81 | 0.82 |  | 0.84 | 0.86 |  | 0.77 | 0.79 |  | 0.77 | 0.79 |  | 0.88 | 0.89 |  | 0.83 | 0.84 |
| Work-Home Conflict | 0.78 | 0.78 |  | 0.80 | 0.80 |  | 0.76 | 0.76 |  | 0.71 | 0.73 |  | 0.86 | 0.86 |  | 0.82 | 0.83 |
| Techno-Ease | 0.84 | 0.85 |  | 0.85 | 0.85 |  | 0.83 | 0.84 |  | 0.81 | 0.84 |  | 0.84 | 0.85 |  | 0.91 | 0.91 |
| Techno-Reliability | 0.86 | 0.86 |  | 0.82 | 0.83 |  | 0.86 | 0.87 |  | 0.82 | 0.82 |  | 0.95 | 0.96 |  | 0.83 | 0.84 |
| Techno-Sociality^a^ | 0.77 | - |  | 0.78 | - |  | 0.61 | - |  | 0.89 | - |  | 0.80 | - |  | 0.85 | - |
| Pace of Change | 0.88 | 0.88 |  | 0.89 | 0.89 |  | 0.88 | 0.88 |  | 0.86 | 0.87 |  | 0.86 | 0.86 |  | 0.82 | 0.82 |
| **Academic Motivation Scale-College** |  |  |  |  |  |  |  |  |  |  |  |  |  |  |  |  |  |
| Intrinsic Motivation-to Know | 0.90 | 0.90 |  | 0.92 | 0.92 |  | 0.88 | 0.99 |  | 0.88 | 0.88 |  | 0.91 | 0.90 |  | 0.88 | 0.89 |
| Intrinsic Motivation-Toward Accomplishment | 0.87 | 0.86 |  | 0.86 | 0.86 |  | 0.81 | 0.81 |  | 0.88 | 0.88 |  | 0.87 | 0.87 |  | 0.88 | 0.88 |
| Intrinsic Motivation-Experience Stimulation | 0.87 | 0.88 |  | 0.87 | 0.88 |  | 0.84 | 0.84 |  | 0.83 | 0.63 |  | 0.89 | 0.90 |  | 0.85 | 0.86 |
| Extrinsic Motivation-Identified | 0.82 | 0.82 |  | 0.81 | 0.81 |  | 0.79 | 0.79 |  | 0.84 | 0.84 |  | 0.84 | 0.84 |  | 0.84 | 0.84 |
| Extrinsic Motivation-Introjected | 0.85 | 0.85 |  | 0.80 | 0.80 |  | 0.87 | 0.87 |  | 0.86 | 0.87 |  | 0.90 | 0.90 |  | 0.83 | 0.83 |
| Extrinsic Motivation-External Regulation | 0.82 | 0.82 |  | 0.84 | 0.85 |  | 0.77 | 0.77 |  | 0.83 | 0.84 |  | 0.80 | 0.80 |  | 0.86 | 0.86 |
| Amotivation | 0.86 | 0.86 |  | 0.85 | 0.85 |  | 0.88 | 0.88 |  | 0.74 | 0.75 |  | 0.88 | 0.87 |  | 0.87 | 0.87 |
| **Hospital Anxiety and Depression Scale** |  |  |  |  |  |  |  |  |  |  |  |  |  |  |  |  |  |
| Anxiety | 0.85 | 0.83 |  | 0.86 | 0.85 |  | 0.83 | 0.82 |  | 0.83 | 0.80 |  | 0.82 | 0.80 |  | 0.84 | 0.82 |
| Depression | 0.75 | 0.75 |  | 0.75 | 0.75 |  | 0.76 | 0.76 |  | 0.67 | 0.67 |  | 0.80 | 0.80 |  | 0.81 | 0.81 |

*Note.* ^a^McDonald’s ω values not available given that the number of items is less than three.
